# Sex Differences in Large Artery Stiffness: Implications for Cerebrovascular Dysfunction and Alzheimer’s Disease

**DOI:** 10.3389/fragi.2021.791208

**Published:** 2021-12-01

**Authors:** Mackenzie N. Kehmeier, Ashley E. Walker

**Affiliations:** Department of Human Physiology, University of Oregon, Eugene, OR, United States

**Keywords:** arterial stiffness, pulse pressure, endothelial cell, cerebrovascular, cognitive impairment, menopause, estrogen

## Abstract

Two in every three Alzheimer’s disease diagnoses are females, calling attention to the need to understand sexual dimorphisms with aging and neurodegenerative disease progression. Dysfunction and damage to the vasculature with aging are strongly linked to Alzheimer’s disease. With aging there is an increase in stiffness of the large elastic arteries, and this stiffening is associated with cerebrovascular dysfunction and cognitive impairment. However, it is unclear how the deleterious effects of arterial stiffness may differ between females and males. While environmental, chromosomal, and sex hormone factors influence aging, there is evidence that the deficiency of estrogen post-menopause in females is a contributor to vascular aging and Alzheimer’s disease progression. The purpose of this mini review is to describe the recent developments in our understanding of sex differences in large artery stiffness, cerebrovascular dysfunction, and cognitive impairment, and their intricate relations. Furthermore, we will focus on the impact of the loss of estrogen post-menopause as a potential driving factor for these outcomes. Overall, a better understanding of how sex differences influence aging physiology is crucial to the prevention and treatment of neurodegenerative diseases.

## Introduction

Advancing age is the biggest risk factor for late-onset Alzheimer’s disease (AD), suggesting that elements of the aging process initiate or contribute to AD. In the United States, two-thirds of patients with AD are females (Alzheimer’s Association, 2013) and the progression from mild cognitive impairment to AD is quicker in females than males (Lin et al., 2015). However, the causes of the increased AD risk in females are not entirely clear. The contribution of the aging vascular system in AD onset and progression is supported by recent evidence (Kapasi and Schneider, 2016). Therefore, sex differences in vascular aging represent a potential source of the greater AD risk in females.

A primary characteristic of vascular aging is the stiffening of the large elastic arteries. This age-related increase in arterial stiffness is related to cognitive impairment and AD, and it is hypothesized that cerebrovascular dysfunction links these phenomena (Iulita et al., 2018). While arterial stiffness increases with age in both sexes, there is a stronger association between arterial stiffness and mortality in females compared with males (Coutinho, 2014). Less is known about sex differences in the relations between arterial stiffness and cerebrovascular dysfunction and cognitive impairment. Sexual dimorphisms in age-related arterial stiffening, and the consequences of this stiffness, may explain the sex differences in AD risk, and potentially identify the need for individualized treatment. The goal of this mini review is to highlight the importance of sex differences in vascular aging and the related onset of cerebrovascular dysfunction and AD. Importantly, we will identify the major gaps in knowledge remaining. The impact of sex differences in vascular aging affects a broad range of neurological diseases. Although this mini review focuses on AD, most of the underlying physiological processes discussed have implications for other neurological diseases.

## Sex Hormones

Sex differences in AD risk are likely driven by sex hormones, genotype (XX vs. XY), and sociocultural factors. In particular, the low estrogen in post-menopausal females is a contributor to vascular dysfunction when compared to pre-menopausal female and/or their male counterparts. Estrogen stimulates genomic and nongenomic cell signaling cascades by activation of estrogen receptors (ER) *α* and *β*, and the G-protein coupled receptor, GPER1 (or GPR30) (Zimmerman et al., 2016; Fuentes and Silveyra, 2019). These receptors are found on vascular cells as well as other cells in the brain (Pau et al., 1998; Morissette et al., 2008). Progesterone and androgens also decrease with age, while follicle stimulating hormone and luteinizing hormone increase (Lee et al., 1988; Morley, 2001). In this review, we will specifically focus on the low estrogen state in post-menopausal females given the preponderance of evidence for its importance.

## Large Artery Stiffness

The stiffness of the large elastic arteries increases with age in both males and females; yet there are important sex differences in the causes and rate of progression of this stiffening. The term large arteries, or large elastic arteries, refers to the aorta and carotid arteries. These large arteries have a very distensible wall and a high content of elastin protein. At young ages, females tend to have more compliant large arteries compared with males, but this trend reverses in old age with older females generally having stiffer large arteries compared with males (Waddell et al., 2001; Berry et al., 2004; Coutinho, 2014). These trends result in females experiencing a more rapid increase in arterial stiffness with aging than males, as found in humans (Lu et al., 2020) and rodents (DuPont et al., 2021). This rapid period of increases in arterial stiffness occurs at ∼55–75 years of age in human females, corresponding to the early post-menopausal period and the reduction of estrogen. Hormone replacement therapy with estradiol typically improves arterial stiffness in post-menopausal females (Scuteri et al., 2001). In summary, age-related increases in large artery stiffness are more rapid in females, likely due to declining estrogen post-menopause.

In general, the sources of age-related large artery stiffening are decreased elastin content, increased elastin fragmentation, increased collagen content and crosslinking, and increased vascular tone (Fonck et al., 2009; Hayashi and Hirayama, 2017). However, most of these mechanisms were studied in males and little is known about the causes of increased arterial stiffness in females. In animal studies, females have age-related increases in large artery collagen content and advanced glycation end-products, contributing to collagen cross-linking (Qiu et al., 2007; DuPont et al., 2021). Estrogen decreases collagen deposition by cultured smooth muscle cells (Natoli et al., 2005), and thus, post-menopausal females may suffer from a loss of the inhibitory actions of estrogens on arterial collagen production. In addition to differences in structural proteins, age-related arterial stiffening in females is caused by increases in arterial tone from a reduction in nitric oxide (NO) bioavailability (Scuteri et al., 2001). Interventions known to improve NO bioavailability also reduce stiffness in post-menopausal females, such as treatment with antioxidants (Moreau et al., 2005) and endothelial NO synthase (eNOS) co-factor tetrahydrobiopterin (Moreau et al., 2012). Furthermore, sympathetic nerve activity increases with age in females and has been related to large artery stiffness, potentially due to increased arterial tone or blood pressures (Harvey et al., 2017; Holwerda et al., 2019). Lastly, signaling by smooth muscle mineralocorticoid receptors contributes to increased age-related aorta stiffening, but the mechanisms appear to be different between male and female mice (DuPont et al., 2021). The causes of sex differences in large artery stiffness have been more thoroughly reviewed by Moreau and Hildreth (Moreau and Hildreth, 2014) and DuPont et al. (2019).

## Blood Flow and Pressure Pulsatility

As large artery stiffness increases, there is greater pulsatility of blood pressure and blow flow (Mitchell, 2018). At young ages, the large arteries are highly compliant and dampen the pulse of blood ejected from the heart. The cerebral vasculature is also protected from highly pulsatile pressure and flow due to a partial reflection of the pressure wave before it reaches the brain. This partial reflection of the pressure wave results from the mismatch of stiffness between the highly complaint aorta and the stiffer muscular arteries (Mitchell, 2018). As the aorta stiffens with age, there is less wave reflection and a higher transmission of pulsatile energy to small arteries, arterioles, and capillaries in the brain (Mitchell, 2018). It is thought that the resulting increased pressure and flow pulsatility in the cerebral vasculature leads to damage and dysfunction (de Montgolfier et al., 2019). While young females have lower cerebral artery blood flow pulsatility compared with young males (Alwatban et al., 2021), this protection does not persist into old age. In fact, the rate of increase in middle cerebral artery blood flow pulsatility with aging is greater in females than in males (Alwatban et al., 2021), corresponding to the more rapid increase in large artery stiffness in aging females. Older females also have less pulsatile dampening between the carotid and cerebral arteries compared with older males (Lefferts et al., 2020), further illustrating a higher transmission of pulsatile energy into the brain of older females. These findings suggest that the female brain at young ages is protected from high pulse pressures, but is exposed to a rapid increase, greater than males, in pulse pressure with aging.

## Cerebrovascular Endothelial Dysfunction

The age-related increase in pulse pressure in the cerebral vasculature is thought to cause endothelial cell dysfunction. The endothelium is an integral regulator of cerebral blood flow and blood brain barrier (BBB) permeability, thus age-related dysfunction of the cerebral endothelium can lead to impairment in the brain. Endothelial cells react to stimuli by releasing several substances that cause dilation or constriction of blood vessels. At the arteriole and capillary level, a properly functioning endothelial layer is needed to coordinate the vascular, immune, and neural cells that comprise the neurovascular unit (Daneman and Prat, 2015). A key function of endothelial cells is to produce NO that signals smooth muscle cells and pericytes for relaxation (Vanhoutte et al., 2017). During aging, decreased NO bioavailability is caused by increased oxidative stress, specifically via the reaction of superoxide with NO (Donato et al., 2015). This reduction in NO bioavailability with aging can lead to an imbalance of vasodilation and vasoconstriction signals and poses a major issue for the tight regulation of cerebral blood flow.

The BBB protects the brain from circulating pathogens and is composed of endothelial cells joined together by tight junction proteins (Daneman and Prat, 2015). The health of endothelial cells, as well as other cells of the neurovascular unit, is important to maintaining a functional barrier. Furthermore, brain endothelial cells can tightly regulate transcytosis, limiting vesicle-mediated movement of solutes in and out of the brain (Daneman and Prat, 2015). Dysfunction of the BBB contributes to AD by allowing the entrance of substances (e.g., neurotoxins, immune cells) that result in increased inflammatory signaling and oxidative stress, stimulating amyloid-*β* (A*β*) production (Sweeney et al., 2018). A dysfunctional BBB will also lead to impaired clearance of A*β* from the brain, and this impaired clearance is thought to be the primary cause of A*β* plaque deposition in AD (Mawuenyega et al., 2010). Thus, age-related dysfunction of endothelial cells contributes to impaired cerebral blood flow and a dysfunctional BBB.

Estrogen acts favorably on the cerebral vasculature by improving the function of endothelial cells (Krause et al., 2006), a phenomenon that is lost post-menopause. The endothelium has widely expressed ER*α*, and binding to this receptor results in increased eNOS expression and activation *via* phosphorylation, leading to greater endothelial dependent vasodilation (Haynes et al., 2000). Estrogen also decreases oxidative stress by reducing mitochondrial superoxide production (Torres et al., 2018) and increasing endogenous antioxidants (Strehlow et al., 2003). In post-menopausal females, there is decreased ERα expression in the vasculature (Gavin et al., 2009) and post-menopausal females have marked impairments in endothelial function compared with pre-menopausal females (Taddei et al., 1996). See Robinson et al. for a more thorough review of this topic (Robison et al., 2019).

The cerebral vasculature appears to be particularly susceptible to the damaging effects of increased large artery stiffness and pulse pressure. High pulse pressures applied to cerebral arteries *ex vivo*, as well as circumferential stress of cultured endothelial cells, leads to increased oxidative stress (Gatti et al., 2008; Raignault et al., 2017; Girão-Silva et al., 2021). Greater large artery stiffness in a rodent model leads to impaired cerebral artery endothelium-dependent vasodilation by increased oxidative stress and decreased NO bioavailability (Walker et al., 2015). Increased large artery stiffness also leads to a more permeable BBB in rodents (Muhire et al., 2019). However, these mechanistic studies have yet to be performed in females. It is reasonable to assume that young females are doubly protected against this phenomenon owing to lower arterial stiffness and the protective effects of estrogens directly on the endothelium. The endothelium of older females may be more susceptible to the negative consequences of large artery stiffness, but this is an area that requires more investigation.

## Cerebral Blood Flow

Cerebral endothelial cell dysfunction will disturb the tight regulation of blood flow in the brain. Young females have greater cerebral blood flow compared with males; however, the declines in cerebral blood flow with aging are greater in females, such that at old ages there are no differences in cerebral blood flow between females and males (Liu et al., 2016; Aanerud et al., 2017; DuBose et al., 2018). More important than global cerebral blood flow is the ability for local blood flow to change in response to stimuli and to be directed to working regions of the brain, indicated by cerebrovascular reactivity. Cerebrovascular reactivity declines with advancing age to a greater extent in females than males, and hormone replacement therapy can preserve cerebrovascular reactivity in post-menopausal females (Kastrup et al., 1998). The sex differences in cerebral blood flow and reactivity with aging, as well as the mechanisms, are extensively reviewed in Barnes and Charkoudian (Barnes and Charkoudian, 2021).

While sex differences in cerebral blood flow and reactivity are extensively investigated, less is known about these in relation to large artery stiffness. The association between large artery stiffness and reduced cerebral blood flow or cerebrovascular reserve has been demonstrated in human subjects, but this was independent of sex (DuBose et al., 2018) or was not analyzed for sex differences (Jefferson et al., 2018). Rodent models of induced large artery stiffness demonstrate the cause-and-effect relation between large artery stiffness and reduced cerebral perfusion (Knutsen et al., 2018; Muhire et al., 2019) but these studies were performed in only male rodents. Thus, a crucial area for future research is to understand the impact of sex and sex hormones on the relation of large artery stiffness and cerebral blood flow regulation, as well as the potential modulation of this relation by other factors.

## Neuropathology

Endothelial dysfunction, BBB permeability, and reduced cerebral blood flow are key mechanisms leading to other pathologies in the brain. For example, large artery stiffness is related to cerebral small vessel disease, a disease that is characterize by hyperintensities, cerebral microbleeds and lacunar infarcts (Mitchell et al., 2011; Poels et al., 2012; Rosano et al., 2013; Hughes et al., 2018; Rensma et al., 2020). Aortic augmentation index, an indicator of arterial stiffness, is also related to white matter hyperintensities in post-menopausal females (Barnes et al., 2017). However, no other studies have examined sex differences in the relation between cerebral small vessel disease and arterial stiffness.

Large artery stiffness is also related to lower brain volumes abnormalities and amyloid-*β* deposition (Mitchell et al., 2011; Hughes et al., 2018). The causative nature of increased large artery stiffness on neurodegeneration and neuroinflammation was demonstrated in rodents (Sadekova et al., 2018). There is a suggestion that these relations between peripheral pulse pressure and neuropathology may have sex differences, as it was found that females had a stronger correlation between brachial pulse pressure and white matter microstructure changes (Reas et al., 2021). Notably, this strong correlation in females is only true early post-menopause, corresponding to the period of more rapid stiffening of the large arteries, and is not found for the group over 75 years of age (Reas et al., 2021). Thus, studies indicate an association between large artery stiffness and neuropathology, but the knowledge of how sex and sex hormones effect these relations is very limited.

## Cognitive Function

Large artery stiffness, and the resultant cerebrovascular dysfunction, will potentially impact the brain, leading to cognitive impairment. The literature regarding sex differences in cognitive function in older adults is inconsistent. This is partly due to sex differences in the specific types of cognitive function that change with age. Older females typically score better on verbal tasks than males, while older males score better on visuospatial and motor coordination than females (Weiss et al., 2003). An important sex difference is that older females experience a more rapid cognitive decline, with the transition from mild cognitive impairment to AD occurring faster compared with age-matched males (Lin et al., 2015). There are numerous studies demonstrating a correlation between greater large artery stiffness and cognitive impairment. While most of these studies controlled for sex in their analyses, none of them report analysis specifically for sex differences in these relations (Hanon et al., 2005; Mitchell et al., 2011; Tarumi et al., 2011; Pase et al., 2016; Meyer et al., 2017; Rouch et al., 2018) except the study by Singer et al. In that study of subjects 70–90 years of age, a relation between large artery stiffness and memory was found in males, but not females (Singer et al., 2013). However, as the rapid progression of arterial stiffness occurs from 55 to 75 years of age in females, this study may have missed the key time for relations in females. In rodents, induced carotid artery stiffening leads to cognitive impairment, but these studies are limited to male rodents to date (Muhire et al., 2019). The sex differences in the relation of arterial stiffness and cognitive decline are likely more complex than just differences in sex hormones. For example, history of pregnancy and childbirth may contribute as hemodynamic properties of the aorta are associated with cognitive function in post-menopausal females, but a history of preeclampsia influences this association for some cognitive abilities (Miller et al., 2020). Therefore, more research is needed to understand how sex may influence the effects of large artery stiffness on cognitive function.

## Perspectives: A Two-Hit Hypothesis for Female Brain Aging and Remaining Gaps in Knowledge

The current hypothesis is that an age-related increase in large artery stiffness and pulse pressure leads to cerebrovascular and cognitive impairment. As the age-related stiffening of the large arteries is slower to progress in males, this may allow time for adaptation of the cerebral vasculature to elevated pulse pressure. In females, post-menopause, there is a more rapid increase in arterial stiffness, and this coincides with the loss of estrogen’s protective effects on endothelial cells. Thus, early post-menopausal females are susceptible to two-hits simultaneously that can lead to cerebrovascular and cognitive impairment, and this may explain the increased AD risk in females.

A few factors have led to the paucity of data regarding sex differences in the effects of large artery stiffness on AD-related outcomes, such as the historical exclusion of females from studies and the treatment of sex as a confounding variable rather than an important contributor to physiology. In addition, ovariectomy is often used to induce a menopause-like state in young rodents matching human surgically induced menopause; however, this is distinctly different from natural human menopause as 1) the effects of estrogen deficiency may impact young and old females differently, and 2) human menopause typically does not have a sudden onset of estrogen loss (Diaz Brinton, 2012). Lastly, differences in the age of subjects may contribute to inconsistencies in the literature, as the rapid increase in arterial stiffness and cognitive decline are typically only found before the age of 75 years in females. Therefore, future studies need to include females in peri- and early post-menopause to understand these key physiological changes.

## Conclusion

**FIGURE 1:**
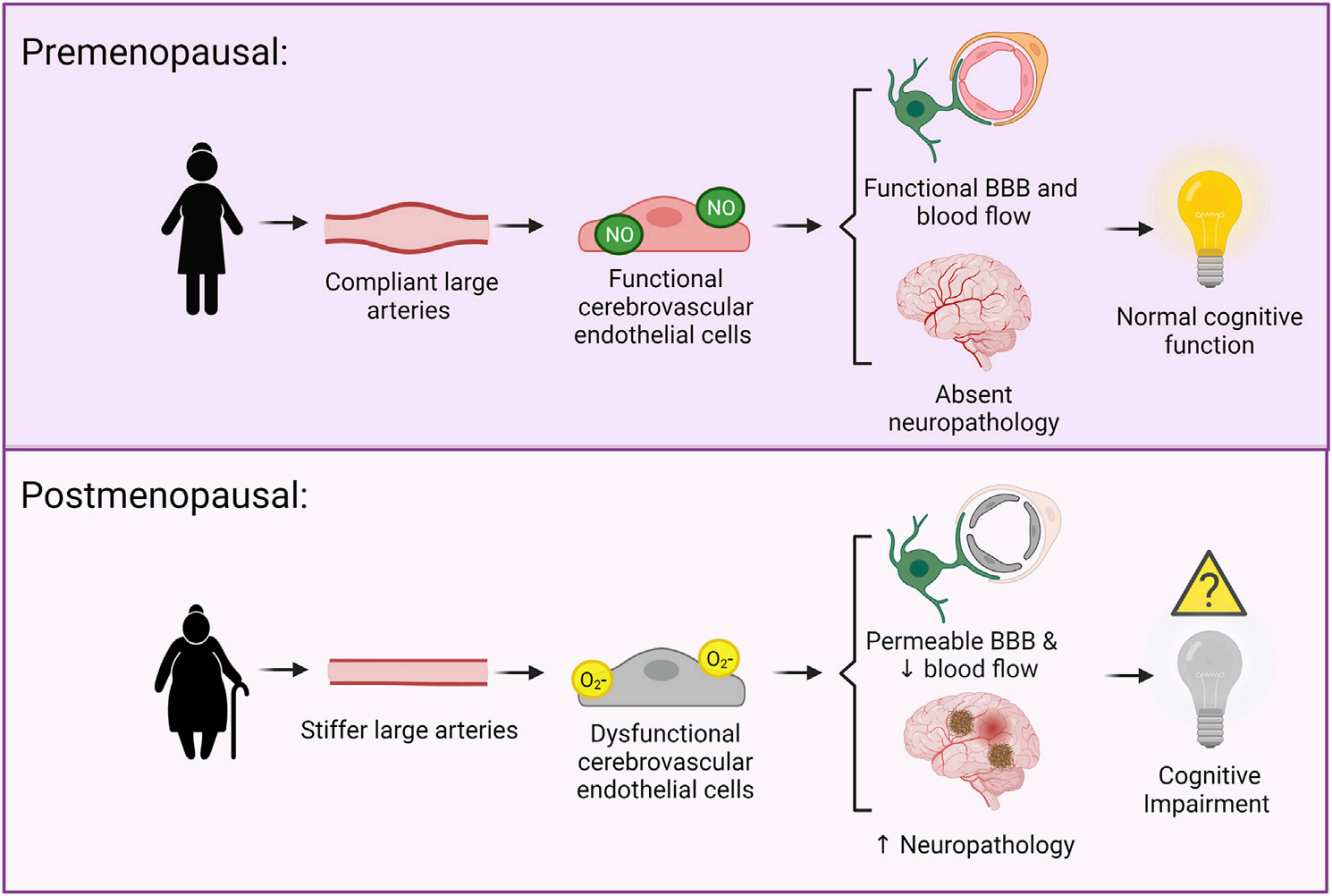
Hypothesized mechanisms linking large artery stiffness and cognitive impairment. Above: In premenopausal females, the large arteries are compliant and cerebral pressure and blood flow pulsatility is low. This is associated with functional cerebral endothelial cells, a functional blood brain barrier, adequate cerebral blood flow, and an absence of neuropathology. Below: In postmenopausal females, there is greater large artery stiffness and higher cerebral pressure and blood flow pulsatility. This is associated with dysfunction of the cerebral endothelial cells, a more permeable blood brain barrier, neurovascular uncoupling, reduced cerebral blood flow, and increased neuropathology.

Age-related increases in large artery stiffness are associated with cerebral endothelial cell dysfunction, reduced cerebral blood flow, neuropathology, and cognitive impairment. As females experience a more rapid increase in large artery stiffness with aging, coinciding with menopause, they could be more susceptible to these damaging effects, and this may explain their increased risk for AD (Figure 1). These deleterious effects of increased large artery stiffness in older females likely contribute to other neurological diseases in addition to AD. Numerous efforts, in both human and animal studies, are needed to close the gaps in knowledge about the effects of vascular aging on the female brain.
